# Fluorescent recognition of Fe^3+^ in acidic environment by enhanced-quantum yield N-doped carbon dots: optimization of variables using central composite design

**DOI:** 10.1038/s41598-020-68390-8

**Published:** 2020-07-16

**Authors:** Mohammed Abdullah Issa, Zurina Z. Abidin, Shafreeza Sobri, Suraya Abdul Rashid, Mohd Adzir Mahdi, Nor Azowa Ibrahim

**Affiliations:** 1 Department of Chemical and Environmental Engineering, Faculty of Engineering, Universiti Putra Malaysia, 43400 UPM Serdang, Selangor Malaysia; 2 Department of Computer and Communications Systems Engineering, Faculty of Engineering, Universiti Putra Malaysia, 43400 UPM Serdang, Selangor Malaysia; 3 Department of Chemistry, Faculty of Science, Universiti Putra Malaysia, 43400 Serdang, Selangor Malaysia

**Keywords:** Chemical engineering, Environmental sciences, Biophysics, Nanoscale biophysics, Analytical chemistry, Chemical engineering, Environmental chemistry, Green chemistry, Organic chemistry, Surface chemistry

## Abstract

A versatile synthetic approach for development of highly fluorescent nitrogen-doped carbon dots (N-CDs) from carboxymethylcellulose in the presence of linear polyethyleneimine (LPEI) has been developed. According to single factor method, central composite design incorporated with response surface methodology matrix was applied to find and model optimal conditions for the temperature (220–260 °C), duration (1–3 h) and LPEI weight (0.5–1.5%). The statistical results show that duration was the most significant parameter for efficient carbonization conversion rate in comparison with temperature and LPEI weight. The reduced cubic model (R^2^ = 0.9993) shows a good correlation between the experimental data and predicted values. The optimal variables were temperature of 260 °C, duration of 2 h and LPEI weight of 1%. Under these conditions, quantum yield of up to 44% was obtained. The numerically optimized N-CDs have an average size of 3.4 nm with graphitic nature owing to the abundant amino species incorporated into the carbon core framework. The blue-green N-CDs possess emission dependent upon the solvent polarity, wide pH stability with enhanced emission in an acidic environment. Impressively, the N-CDs show long-shelf-life for up to 1 year with no noticeable precipitation. The N-CDs were able to recognize a high concentration of Fe^3+^ ions with a detection limit of 0.14 μM in acidic solution owing to the special coordination for Fe^3+^ to be captured by electron-donating oxygen/ amino groups around N-CDs. Moreover, the N-CDs can also be used as a new kind of fluorescent ink for imaging applications.

## Introduction

Carbon dots (CDs) are the latest member of fluorescent carbon nano-sized family. Typically, CDs are nearly spherical-shaped nanoclusters with sizes of less than 10 nm and consist of amorphous or crystalline cores with sp^2^ carbon atoms^1^. Since the first established work^2^, CDs have attracted a considerable focusing in the fields of wastewater treatment, photocatalysis, bioimaging, cancer therapy and chemical sensing^3,4^. This is owing to their feature of having outstanding optical properties, including excellent biocompatibility, tuneable photoluminescence, negligible toxicity, ease of production and resistance to photobleaching in comparison to QDs counterparts^5–7^. It is well known that QDs, for instance, tend to be decayed in the biological environment leading to a serious toxicity concern^8^.

The synthesis of CDs can be broadly categorized into two modes: the top-down and bottom-up synthetic routes. Arc discharge, laser ablation and chemical oxidation are among the top-down techniques, which involve cleaving bulk-sized carbon precursors, such as graphite and carbon nanotubes, into nano-sized materials. Meanwhile, the bottom-up approach usually employed to prepare CDs from carbon-rich resources, including citric acid, glucose and chitosan through microwave-assisted pyrolysis, ultrasonic and hydrothermal carbonization^9^. In comparison to top-down technique, bottom-up routed offers rapid, low-cost and eco-friendly. Among all the available methodologies, hydrothermal carbonization (HTC) process is one of the most preferred synthesis routes in preparing CDs due to its versatile, short duration, cost-effectiveness and ability to use extensive variety of accessible carbon precursors.

To synthesize CDs, the selection of a suitable starting material plays a significant role. A perfect carbon source for green CDs production should be (1) available worldwide, (2) not be in direct competition with essential food production and (3) cost-effective. A summary of several sustainable precursors used for developing CDs via HTC route is listed in Table S1. As shown in the Table, the usage of small bioresources resulted in low QY^10–15^. On the other hand, doping CDs with various non-metal moieties particularly N dopants gained a considerable scientific focusing as it can enhance the fluorescent (PL) emission through tuning the upward shift of the Fermi level and introduce new energy states related to the N-functionalities^7,16^. Despite the spectacular optical characteristics of nitrogen-doped CDs (N-CDs), most of the reported works in the literature displays some shortcomings, including time consumption^17–23^ and low QY of N-CDs^17,18,21–23^, thus restricting their practical applications. Hence, the need to find alternative agents that can play an effective role to manipulate the intrinsic properties and improve the optical performances of N-CDs, and even produce unexpected phenomena and applications is highly desirable.

Most of previous studies employed one factor at a time (OFAT) to improve the optoelectronic properties of N-CDs and hence optimize the response of interest^22,24^. In spite of high PL efficiency that obtained, the major drawbacks associated with these traditional methods are as follows: (1) time consuming; (2) high expense of reagent and materials consumption; and (iii) inability to evaluate the effectiveness and interactive among of tested variables on the response. The main goal of the experimental design is to study the interactions among the significant variables, optimize the response and provide statistical model with at least experiments^25,26^. In this concept, very limited efforts on the RSM applications in the optimization synthesis processes of N-CDs were carried out^27–29^ as presented in Table S2. Central composite design (CCD) incorporated with response surface methodology (RSM) is considered to be the most preferable and practical design for optimization applications of N-CDs. Thus, more statistical optimization investigation to explore the best fluorescence QY conditions is highly desirable.

Heavy metal ions are one of the most poisonous contaminants that cause severe environmental and health threats^30^. Up to now, one of the widespread issues are related to the presence of metallic compounds at high doses in water systems^31^. In particular, the ferric ion (Fe^3+^) commonly exists in the human body and environment. The excess of ferric can induce an acknowledged risk of diseases, such as liver damage, kidney failure, or even death^8,32^. For these reasons, it would be sensible to develop rapid analytical tools for the monitoring of Fe^3+^ ions in aqueous system.

**Figure 1:**
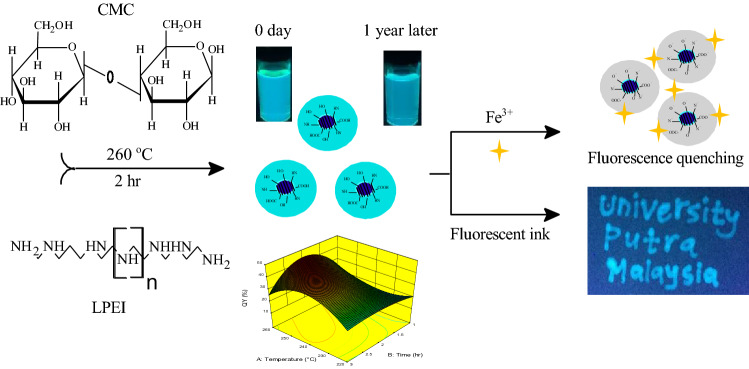
Schematic illustration of N-CDs synthesis and its application for the detection of Fe^3+^ and fluorescent ink.

In this context, we report a facile route of highly fluorescent N-CDs for fluorometric sensing of Fe^3+^ (Fig. 1). To determine preliminary range of variables in the N-CDs process, single factor approach was carried out and used for modelling the experimental data. A detailed determination of QY is presented by measuring the absorption and PL emission spectra for each experimental run. Response surface methodology combined with faced-centered CCD was applied to study the influence of temperature, time and LPEI weight on the QY of N-CDs and hence maximize the response. The as-formed N-CDs suspension with the highest QY was further analyzed by various analytical methods, and their PL stability as a function of solvent, pH, and long-term storage time were examined. In addition, N-CDs were employed as nanoprobes for Fe^3+^ sensing and successfully applied in real water. Furthermore, these N-CDs were used as invisible ink for security purposes.

## Experimental

### Materials

CMC of oil palm empty fruit bunch was purchased from Waris Nove Company, Malaysia. Analytical-grade of LPEI (Mn ~ 5,000) and quinine sulfate were obtained from Sigma-Aldrich (USA). Deionized water (DI) has been used throughout all the solution preparation.

#### Synthesis of N-CDs

0.1 g of fine CMC and a suitable amount of LPEI were dispersed into 25 ml of DI water by means of ultrasonication to ensure uniform dispersion. The mixture was then transferred to a 50 ml Teflon-lined stainless steel reactor, sealed and heated at 260 ^◦^C for 2 h in an oven. The obtained solution was cooled down naturally and then centrifuged at 10,000 rpm for 12 min to get free-dark carbonaceous material. The final solution was purified by vacuum filtration (0.22 µm) to remove the precipitate and subjected into a dialysis membrane (1 kDa) to completely eliminate salt ions.

### Characterization methods

Transmission electron microscopy (TEM) and high-resolution TEM (HRTEM) readings were performed using A Tecnai G2 F20 electron microscope, with an acceleration voltage of 200 kV. The Fourier transform infrared (FTIR) (Thermo Nicolet FTIR spectrometer of 4 cm^−1^ resolution) was carried out with KBr as a standard. X-ray photoelectron spectra (XPS) (Physical Electronics PHI 5,400 spectrometer, Mimos Semiconductors, Kuala Lumpur, Malaysia) were reacorded by Al-Ka radiation (${\text{h}{\upnu }} = 1486.6$ eV). Zeta potential study was obtained using Zetasizer Nano ZS (Malvern, UK). UV–Vis spectra of all samples were recorded using Shimadzu UV-1800 Spectrophotometer. Fluorescence studies were measured in quartz cuvettes with 1 cm path length using LS 55 Fluorescence Spectrometer (PerkinElmer, USA). Quinine sulfate in 0.1 M H_2_SO_4_ (QY = 54%) was used as a reference fluorophore. Prior to absorption and PL spectrum measurements, all the as-prepared solutions and reference samples were diluted to a concentration of 10^−5^ M in DI water for obtaining maximum optical density of less than 0.5 at an excitation wavelength of 350 nm. This step has an advantage of minimizing re-absorption effect caused by the interaction between the fluorophore molecules that might contaminate the accuracy of the readings. *The* QY were then calculated by comparing both absorption and PL emission of all aqueous N-CDs with that of quinine sulfate using the following formula^18,33–37^:

1 $$ {\text{QY}} = Q_{R} \frac{I}{{I_{R} }} \frac{{OD_{R} }}{OD} \frac{{\eta^{2} }}{{\eta_{{R^{2} }} }} $$

where (*I*) is integrated intensity, ($OD$) optical density and ($\eta$) is refractive index. The subscript $R$ refers to the reference fluorophore of known QY. Origin 9.0 (OriginLab Corporation, Northampton, MA, USA) was used for PL curve fitting and Design-Expert 10.0 (stat Ease inc, Minneapolis, USA) were carried out for statistical analysis.

### Experimental matrix and optimization via RSM

Response surface methodology (RSM), a mathematical design tool used to fit the experimental data in order to maximize the response. In this work, RSM incorporated with central composite design (CCD) was applied to find the optimum value of the PL QY. Prior to designing the experiments, one factor at a time (OFAT) approach was performed to determine the operational range of each variable. On the basis of OFAT method, the significant variables were selected as follows: synthesis temperature (X_1_), reaction duration (X_2_), and LPEI weight (X_3_). Each variable was investigated using faced-centered CCD at three practical levels namely − 1, 0, and + 1 representing the lowest, center, and highest, respectively to obtain the overall design matrix. Table 1 represents the range and levels of each independent variable. CCD involves 2^3^ factorial design with the total of 18 experiments, including 8 factorial runs, 6 axial runs and 4 replicates at the central point for estimation error. The matrix of CCD along with the experimental values of QY are given in Table 1. In this study, the mass of CMC and the ratio of water/LPEI were fixed to be 0.1 g and 40, respectively, throughout all the designing experiments. More details on the evaluation of statistical optimization can be found in the supplementary materials.

**Table 1:** Actual and coded range and levels of significant variables.

Symbol	Variable	Range and level		
		− 1	0	+ 1
X_1_	Temperature (°C)	220	240	260
X_2_	Duration (h)	1	2	3
X_3_	LPEI weight (%)	0.5	1	1.5

### Detection of Fe^3+^ ions

The nanoprobe acidic suspension was prepared as follows: 75 μM of N-CDs solution was taken in a 50 ml standard measuring flask and tuned up to the mark by hydrochloric acid (pH 3). The feasibility of N-CDs for sensing Fe^3+^ was performed at different concentrations (from 0 to 400 μM) of Fe^3+^. 2 ml of a particular concentration of Fe^3+^ was added into 2 ml of the probe and the PL spectrum was measured after incubation for 1 min with excitation peaks at 350. The limit detection (LOD) of the N-CDs towards Fe^3+^ in water was estimated using the following equation^32^:

2 $$ {\text{LOD}} = \frac{3S}{b} $$

where *S* represents the standard deviation of the blank signal and *b* refers to the slope of the calibration curve.

To test the selectivity study, various metal ions, including Ag^+^, Fe^3+^, Fe^2+^, Pb^2+^, Mg^2+^, Ca^2+^, Cd^2+^, Zn^2+^, Mn^2+^, Hg^2+^, Ni^2+^, Co^2+^, Li^+^, Ba^2+^, K^+^ and Al^3+^, were applied to detect the variation of N-CDs PL emission using a similar manner described above.

### Fluorescent ink preparation

A traditional sketch pen was obtained from commercial market. The internal part washed several times with DI to eliminate the residue of the ink and dried in an oven at 80 °C for 1 h. After that, the drying part was immersed in the concentrated aqueous dispersion of N-CDs (1 mg ml^-1^), left to settle for 10 min and kept for subsequent experiments.

## Results and discussion

One-step hydrothermal carbonization treatment (HTC) was used to fabricate fluorescent N-CDs from EFB Carboxymethylcellulose and LPEI as the carbon and nitrogen sources, respectively. During carbonization process, the carbonaceous species from EFB Carboxymethylcellulose gets oxygenated, which could provide the elemental and structural basis for the formation of CDs^19^. At the same time, the existence of LPEI can enhance the optoelectronic properties of carbon dots through surface passivation/ N- atom incorporation that increases the upward shift of the Fermi level and electrons in the conduction band^16^.

### Preliminary synthesis study

**Figure 2:**
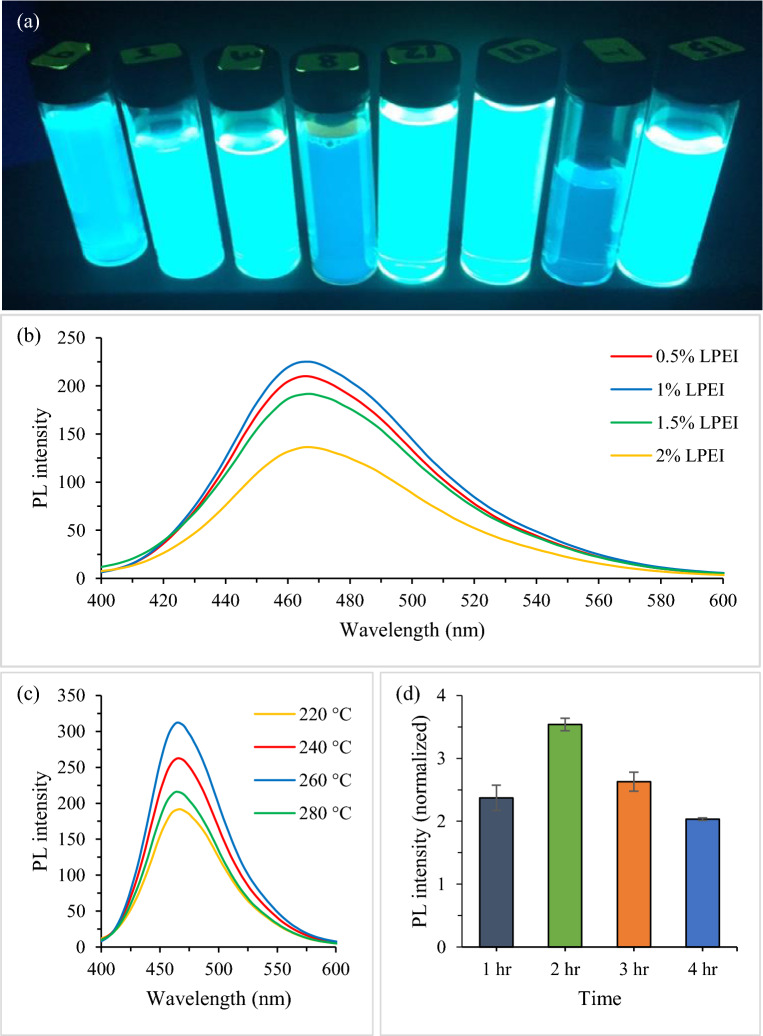
Typical photo of the obtained N-CDs solutions irradiated with UV light at 365 nm (**a**). Preliminary single factor synthesis experiments; PL spectra of N-CDs as a function of (**b**) LPEI weight, (**c**) temperature and (**d**) duration (*λ*ex = 350 nm, *λ*em = 465.5 nm).

Given that the range of influential parameters tends to vary with respect to the starting materials and method used, preliminary experiments were carried out to determine the operational range of each parameter. As shown in Fig. 2a, different range of blue-green color dispersion were obtained while varying the synthesis variables, indicating the formation of N-CDs^16^. This emission variation of N-CDs suggests that temperature, synthesis duration and LPEI weight directly alter the degree of carbonization process and tune the final photophysical properties. It is believed that the bright emission suspensions can be formed due to the sufficiency of carbon nuclear growth. Meanwhile, the formation of transparent solutions (Fig. 2a) confirm that only polymerization reaction of CMC occurs as a result of low temperature-short time process.

While keeping other parameters constant (240 °C, 2 h), the weight of LPEI was varied and measured using PL spectra, as presented in Fig. 2b. It is noticeable that the fluorescence intensity increases with the increased LPEI weight and reaches the maximum when the LPEI weight is at 1%, in which a further increase of LPEI causes obvious emission reduction. Thus, 1% of LPEI weight was chosen as optimal value for subsequent experiments.

The influence of synthesis temperature on the fluorescence spectra is shown in Fig. 2c. Temperature was varied while keeping other synthesis variables constant (2 h, 1% LPEI). It can be seen that the optimal reaction temperature is at 260 °C. Further increase or decrease of temperate has a negative effect on the fluorescence intensity. Therefore, at optimal temperature and LPEI weight, the reaction time was varied and investigated as shown in Fig. 2d. Two hours of synthesis time was found to be the best, in which further increase or decrease lead to significant PL reduction. Based on the OFAT method, the practical levels of significant variables were as follows: temperate (220–260 °C), time (1–3 h), and 0.5–1.5% LPEI weight. These practical ranges of preliminary testing were selected for designing the experiments using RSM.

### QYs determination based on the optical properties of N-CDs

**Figure 3:**
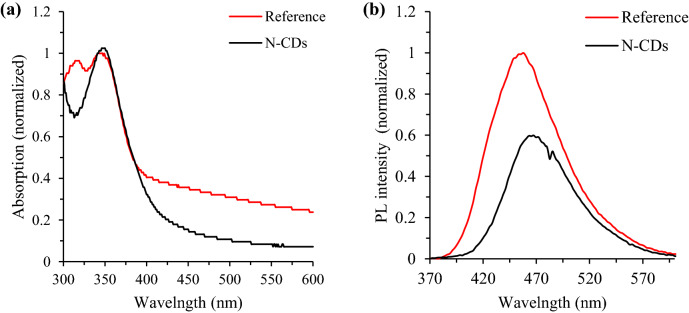
Absorption spectra (**a**) and PL spectra (**b**) of Run #2 for the reference and N-CDs solution at excitation wavelength of 350 nm.

Quinine sulfate was selected as a fluorophore reference due to its wide emission region^38^. The absorption and PL spectra corresponded to Run #2 are shown in Fig. 3a,b. The OD of quinine sulfate and N-CDs solution were kept at 0.033 and 0.04, respectively. The absorption peak is observed at about 350 ± 3 nm (Fig. 3a)—a typical peak for all N-CDs runs. The PL emission for the quinine sulfate and N-CDs solution was centered at 457 and 465.5 ± 3 nm, respectively when both excited at 350 nm (Fig. 3b). Similar PL emissions were also referring to all of the N-CDs runs.

The above-mentioned optical results were facilitated for the QYs determination of all as-synthesized N-CDs. Taking Run #2 as an example, spectroscopic data (recorded in Table 2) were used to calculate the PL QY according to Eq. (1). Prior to QY determination, the PL peak has been integrated for obtaining the corrected area under the peak. Similar procedure was also applied to compute the PL QY of other runs based on the spectroscopic data illustrated in Table S3, in which the ratio of refractive index of each run to the refractive index of the reference ($\eta / \eta_{R}$) were kept to be 1.

**Table 2:** Quantum yield of Run #2.

Sample	Integrated emission intensity (I)	Optical density at 350 nm (OD)	Quantum yield (QY)
Quinine sulfate	35,828.9	0.033	54 (known)
N-CDs sample	16,550.5	0.04	20.58

### RSM design and model fitting

The experimental results in terms of PL efficiency of QY obtained from 18 runs of CCD design are shown in Table 3. The experimental data were fitted to various regression models, including linear, 2F, quadratic and cubic (Table 4). Quadratic model was selected by the software as the recommended model., whereas, the cubic model was considered to be aliased on the basis of low P-value (< 0.05) and high value of F. However, the RSM results show that the PL QY variations could be followed by cubic model with the elimination of some insignificant variables for determining insignificant lack-of-fit and thus fitting the observed data.

**Table 3:** CCD design matrix for the three independent variables.

Run	X_1_	X_2_	X_3_	QY (%)
	Temperature (^°^C)	Duration (h)	LPEI weight (%)	
1	220 (− 1)	1 (− 1)	0.5 (− 1)	13.98
2	260 (+ 1)	1 (− 1)	0.5(− 1)	20.58
3	220 (− 1)	3 (+ 1)	0.5 (− 1)	17.51
4	260 (+ 1)	3 (+ 1)	0.5 (− 1)	18.63
5	220 (− 1)	1 (− 1)	1.5 (+ 1)	12.87
6	260 (+ 1)	1 (− 1)	1.5 (+ 1)	15.69
7	220 (− 1)	3 (+ 1)	1.5 (+ 1)	15.65
8	260 (+ 1)	3 (+ 1)	1.5 (+ 1)	18.01
9	220 (− 1)	2 (0)	1 (0)	25.3
10	260 (+ 1)	2 (0)	1 (0)	44
11	240 (0)	1 (− 1)	1 (0)	15.66
12	240 (0)	3 (+ 1)	1 (0)	39.23
13	240 (0)	2 (0)	0.5 (− 1)	35.16
14	240 (0)	2 (0)	1.5 (+ 1)	26.47
15	240 (0)	2 (0)	1 (0)	38.2
16	240 (0)	2 (0)	1 (0)	38.6
17	240 (0)	2 (0)	1 (0)	37.9
18	240 (0)	2 (0)	1 (0)	37

**Table 4:** Statistical parameters of the polynomial models.

Model	*F*-value	*P* value
Linear	0.58	0.6402
2-factor interaction	0.014	0.9976
Quadratic	12.69	0.0021
Cubic	210.57	< 0.0001

The relationship between the observed QY and independent variables using reduced cubic model can be expressed by the following equation:

3 $$ {\text{QY }}\left( {\text{\% }} \right) = 38.01 + 9.35{ }X_{1} + 11.79{ }X_{2} - 0.74{ }X_{{1{ }}} X_{2} - 3.44{ }X_{1}^{2} - 10.56{ }X_{2}^{2} - 7.28{ }X_{3}^{2} + 0.63{ }X_{1} X_{2} X_{3} - 10.95{ }X_{1}^{2} X_{2} + { }3.28{ }X_{1}^{2} X_{3} - 7.74{ }X_{1} X_{2}^{2} $$

where the negative and positive coefficient values indicate the negative and positive correlation between independent variables and the predicted QY, respectively.

ANOVA was applied to evaluate the quality and significance of the reduced cubic model, (Table 5). The values of *p* and *F* are < 0.0001 and 422.52, respectively, demonstrated that the model is greatly significant. The lack-of-fit of the observed QY is insignificant as the *P* value = 0.7134, suggesting the good predictability of the model^23^. The values of determination coefficient R^2^ = 0.9993 also confirm the great fitting, in which only 0.07% of the total QY variation was not described by the model. The high value of adjusted R^2^ (0.9969) indicated the significance of the developed model.

**Table 5:** ANOVA and regression coefficients for reduced cubic model.

Source	Sum of squares	DF	Mean Square	*F*-value	*P*-value	
Model	2008.89	13	154.53	422.52	< 0.0001	Significant
X_1_-temperature	174.85	1	174.85	478.07	< 0.0001	
X_2_-duration	277.77	1	277.77	759.49	< 0.0001	
X_3_-LPEI weight	37.76	1	37.76	103.24	0.0005	
X_1_ X_2_	4.41	1	4.41	12.06	0.0255	
X_1_ X_3_	0.81	1	0.81	2.21	0.2117	
X_2_ X_3_	1.55	1	1.55	4.23	0.1087	
X_1_^2^	32.15	1	32.15	87.90	0.0007	
X_2_^2^	307.31	1	307.31	840.26	< 0.0001	
X_3_^2^	143.59	1	143.59	392.61	< 0.0001	
X_1_ X_2_ X_3_	3.15	1	3.15	8.61	0.0426	
X_1_^2^ X_2_	191.84	1	191.84	524.54	< 0.0001	
X_1_^2^ X_3_	17.27	1	17.27	47.21	0.0024	
X_1_ X_2_^2^	95.79	1	95.79	261.91	< 0.0001	
Residual	1.46	4	0.37			
Lack of fit	0.075	1	0.075	0.16	0.7134	Insignificant
Pure error	1.39	3	0.46			
Corrected total	2010.35	17				
R^2^		0.9993	Standard deviation		0.6	
Adjusted R^2^		0.9969	Mean		26.14	
Predicted R^2^		0.9462	CV (%)		2.31	
Adequate precision		58.168	PRESS		108.12	

The significance of each regression variable was assessed by *p*-value. In this study, the two independent variables of X_1_ and X_2_ are greatly significant factors with *P* < 0.0001. In addition, the second-order effect such as X_1_^2^, X_2_^2^ and X_3_^2^ are also significant as *P* < 0.05. Moreover, only the interaction between X_1_ and X_2_ were significant on the PL QY of N-CDs. Furthermore, all the cubic interactions such as X_1_X_2_X_3_, X_1_^2^X_2_, X_1_^2^X_3_ and X_1_X_2_^2^ were found to be significant.

The validity of the developed model was also confirmed by the coefficient of variation (CV, %), precision residual sum of square (PRESS) and adequate precision (AP). The values of CV, PRESS and AP (shown in Table 5) were 2.31%, 108.12 and 58.168, respectively, reflecting the acceptable reproducibility and fitness of the proposed model.

**Figure 4:**
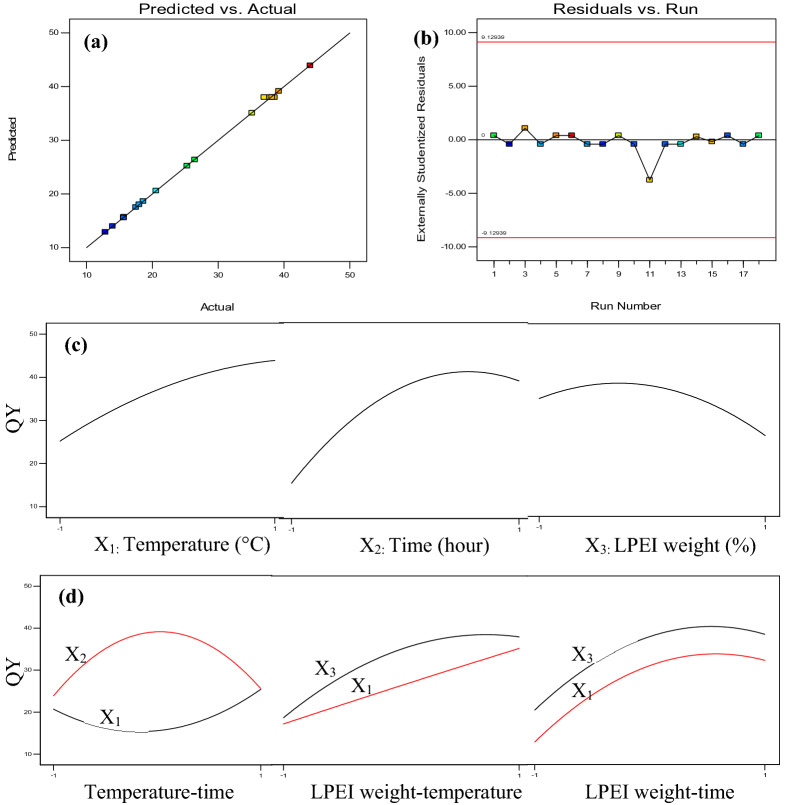
Diagnostic plot for developed model adequacy showing (**a**) actual response versus predicted response for QY and (**b**) studentized residuals versus run number. The main effect of (**c**) temperature, duration, and LPEI weight and (**d**) their binary interactions on the QY of N-CDs obtained from CMC and LPEI.

The relationship between experimental values and the predicted data of each response obtained from Eq. 2 is shown in Fig. 4a. It can be clearly seen that the predicted values are very close to the values of the QY obtained from experiments, with a standard deviation of 0.6 (shown in Table 5). This similarity of the predicted and actual QY justifies the effectiveness of the proposed model. The result of residual plot was also satisfactory (Fig. 4b) as the experimental data were between acceptable range, suggesting the adequacy of the model in calculating the QY at different synthesis conditions.

### Effect of variables on the PL QY

The main influence of temperature, duration, and LPEI weight and the effect of their interactions on the QY are shown in Fig. 4c,d. According to RSM design, the QY of each variable was determined by changing the values of the parameters from the lowest (− 1) to the highest (+ 1) level while the other two variables kept constant at their central level. In case of binary interaction, similar manner for the first factor was performed while the second parameter was held within the level range of − 1 and + 1. Based on Fig. 4c, it can be said that all the factors have synergetic effects on the QY. Increasing temperature leads to the obvious increment of QY. Additionally, when the time ranges between 1 and 3 h, the QY rapidly increases and then decreases, reaching an optimum QY of 41.3% at 2.5 h. Moreover, 0.9% of LPEI weight was found to be sufficient for obtaining the highest QY (~ 38%), in which further increasing of LPEI weight causes significant reduction of PL emission and hence decreases the QY. The above results suggest that time is the most influential factor on the QY.

Figure 4d represents the binary interaction between parameters. It is noticeable that only the interaction of temperature–time is significant, in which the temperature and time were inversely related. In other words, when the synthesis time is short, longer temperature is more favorable to obtain higher QY of N-CDs. The graphs in other interactions are almost parallel, suggesting that no significant influence occurred to the first factor upon alteration the level of second variables.

**Figure 5:**
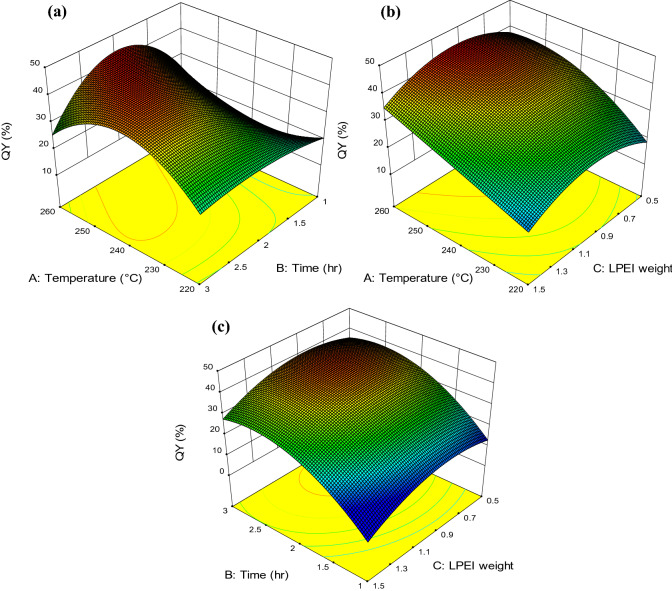
Response surfaces plot obtained based on the reduced cubic model of QY% against the combined effect of (**a**) duration and temperature, (**b**) LPEI weight and temperature, c) LPEI weight and duration.

To get a clear understanding of the relationships between the independent variables and the QY, Eq. (3) was used to facilitate plotting of three-dimensional surfaces representing the response on the Z-axis with any two synthetic parameters. Two parameters were varied within the selected experimental range while keeping the third-factor constant at its center point values (coded level: 0). The effect of both temperature and duration on the QY of N-CDs is represented in Fig. 5a. It can be said that the best range for producing the highest QY was in the range of 240–260 °C for 2 h of synthesis duration. Further increase or decrease in these parameters (especially time) had a negative effect on the QY. The lowest QY was synthesized when both the temperature and duration were at their lowest. This attribute to the reason of only polymerization reaction may occur and consequently the CMC was not completely carbonized. In addition, the combination of high temperature and long duration was found to have negative effect on the QY of N-CDs. This negative effect is again can be explained by the possibility of undesirable reactions could be occurred at high reaction temperature and time. It is believed that when the carbonization degree is too high, N-CDs tend to be aggregated and result in the decline of PL intensity^39,40^.

Figure 5b shows the interaction effect of both temperature and LPEI on the QY of N-CDs at 2-h duration. When the temperature at 220 °C, LPEI shows its lowest effect on the QY, while it has a positive effect when the temperature is increased to up to 260 °C. The possible reason is while LPEI mass increased, much more nitrogen atoms penetrate the crystalline sp^2^ core and disorders the hexagonal ring structure, introducing emissive trap states and thus improving the QY^41^. Figure 5c represents the effect of reaction duration and LPEI weight on the QY at 240 °C. It has been reported that long duration (up to a specific range) can increase the surface oxidation and hence introducing more surface defects on the shell of N-CDs^7^. Based on the results, we can conclude that synthesis duration plays a vital role in the final QY of N-CDs, and followed by the temperature and LPEI weight. It can be noticed that the significance of duration in HTC process become obvious since at rising the temperature to 260 ºC at shortest duration of 1 h resulted in low QY.

### Optimization of variables

The contour plot of reduced cubic design (Fig. S1) shows that the optimum values of the significant variables on the QY are temperature (260 °C), duration (2 h) and LPEI weight (1%). The value of QY under optimal conditions can be seen in Table 6. The synthesis of N-CDs under the optimal conditions resulted in a relatively high QY of 44%. The result validated the model as both the experimental and predicted values were quite similar (QY_pred_ 43.92%). This indicates the suitability of developed reduced cubic model to predict the best conditions within specific ranges in the tested variables.

**Table 6:** Optimum condition derived by the response surface methodology for synthesis of high quantum yield of N-CDs from EFB CMC and LPEI.

Optimum condition			QY (%)		
X_1_ (°C)	X_2_ (h)	X_3_ (%)	Actual	Predicted	Relative error
260	2	1	44	43.92	0.085

It is worth noting that the obtained QY of the present work in a considerably short synthesis duration is much higher than values previously reported for biomass based CDs in the presence of different nitrogen species (Table S1)^19–23^. This suggest LPEI as a great competitor for replacing the traditional N doping agents.

### Morphology and composition of N-CDs

**Figure 6:**
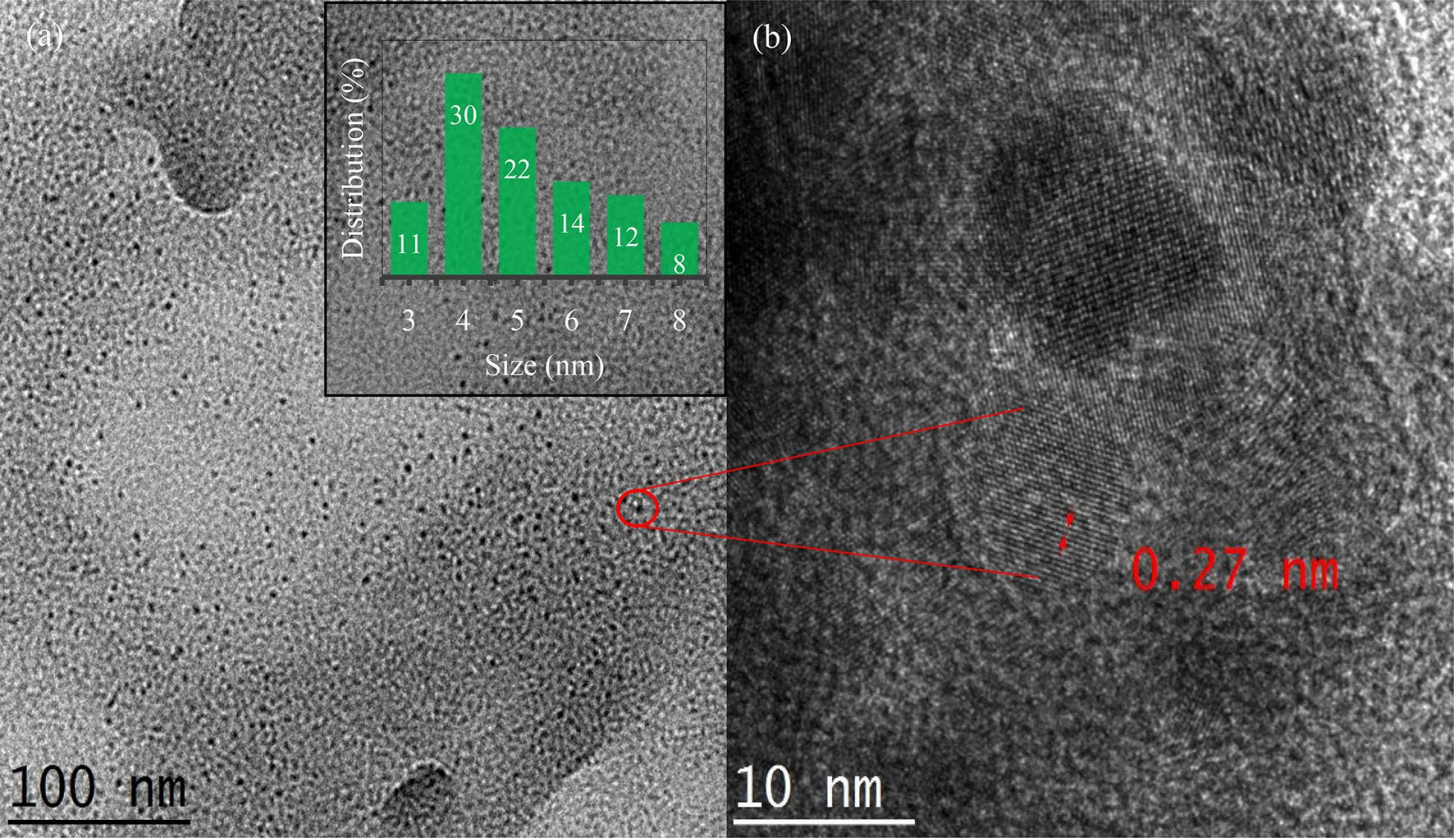
(**a**) TEM image of N-CDs and (**b**) HRTEM image. The inset shows particle size distribution.

The surface morphology of the optimized N-CDs sample was analyzed via TEM and HRTEM. As shown in Fig. 6a, N-CDs are the quasi-spherical shape and separated well from each other. The particle size distribution histogram (inset of Fig. 6a), which was obtained from one hundred nanodots shows that N-CDs have a size distribution of 3–8 nm with an average size of 3.4 nm. HRTEM analysis (Fig. 6b) confirms the graphitic core nature of the obtained N-CDs with an interplanar distance of 0.27 nm, which agrees well with previous reports^42–44^.

**Figure 7:**
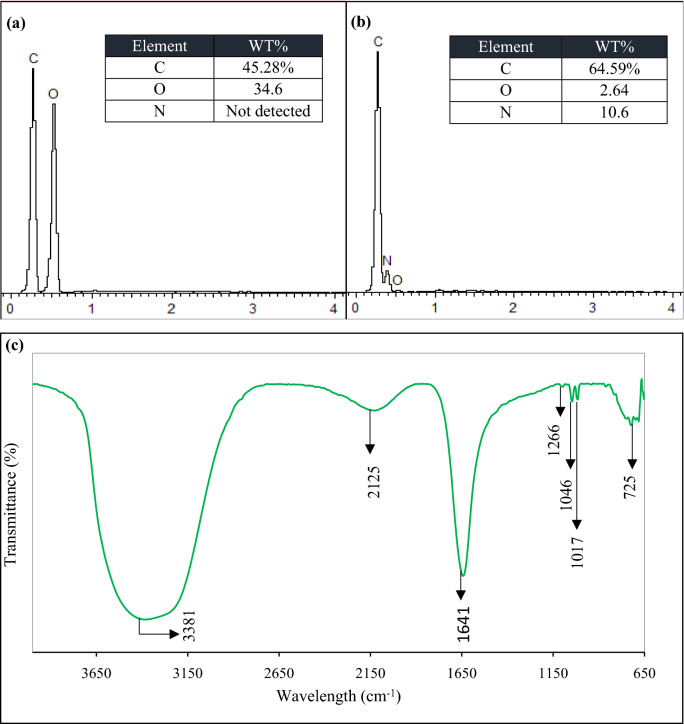
EDS spectrum showing (**a**) raw CMC and (**b**) N-CDs. (**c**) FT-IR spectrum of N-CDs. Table in the inset represents the elemental ratios.

To gain insight into the surface structure of N-CDs, the raw CMC and the obtained N-CDs were characterized using EDS as shown in Figs. 6b and  7a. It is noticeable that the carbon content increases from ~ 45% to ~ 64%, while the oxygen contents are remarkably reduced after HTC treatment of CMC. This indicates that aromatization reaction takes place with the elimination of O containing species. The existence of nitrogen contents with an atomic ratio of ~ 19% after HTC treatment of CMC (Fig. 7b) confirms the excellent incorporation degree of amino moieties into the final surface domains of N-CDs^16^.

FTIR spectra was conducted to better understanding the surface chemical composition of N-CDs. As represented in Fig. 7c, absorption peak at 3,381 cm^-1^ is attributed to stretching vibration of N–H/O–H. Meanwhile, peaks centered at 2,125 cm^-1^ and 725 cm^-1^ corresponded to C–H stretching and bending vibrations, respectively^19,45,46^. The stretching vibration at 1641 cm^-1^ is assigned to C= O, confirming the formation of carboxylic acid groups^47^. Moreover, the peak at 1,266 cm^-1^ is attributed to C–N. The existence of N–H and C–N species confirms the incorporation of LPEI into the final structure of N-CDs through successive dehydration and passivation processes^35,48^. Furthermore, the bending vibration of C–O and C–O–C centered at 1,046 and 1,017 cm^-1^, respectively, proving the formation of organic oxidized groups^4,47^. Generally, the existence of the above functional groups provides great water dispersibility and photostability of these nanoparticles without any further surface modification^7^.

Further confirmation about the binding occurrence and chemical structure of N-CDs was performed using XPS spectrum. As shown in Fig. S2a, the full scan XPS spectrum shows three typical peaks: C_1_s (285 eV), N_1_s (397 eV), and O_1_s (531 eV), which suggest that the N-CDs composed of carbon, nitrogen, and oxygen. The high-resolution spectrum of C_1_s (Fig. S2b) can be separated into four main peaks at 284.6, 285.5, 287.4, and 287.9 eV ascribed to the four states of carbon bonds (C–C, C–N, C–O and C=O respectively). The N_1_s spectrum (Fig. S2c) shows three peaks at 398.8, 399.7 and 400.8 eV, associated with graphitic N, pyridinic N and N–H respectively. This indicates that N has been successfully incorporated into the framework of N-CDs in different modes. The O_1_s band (Fig. S2d) presents two peaks at 530.7 and 531.9 eV for O–H and C=O, respectively. Zeta ($\xi$) potential test (Fig. S3) of the obtained N-CDs solution was found to be − 8.71 mV, indicating the negatively-charged surface of N-CDs. $\xi$ potential study is well consistence with the EDS, FTIR and XPS data, which confirm the formation of multiple containing species like hydroxyl, carboxyl, carbonyl and amino groups around N-CDs and the successful incorporation of N moieties into the sp^2^- conjugated framework of N-CDs.

### Effect of solvent, pH and 1-year aging conditions on the PL stability of N-CDs

**Figure 8:**
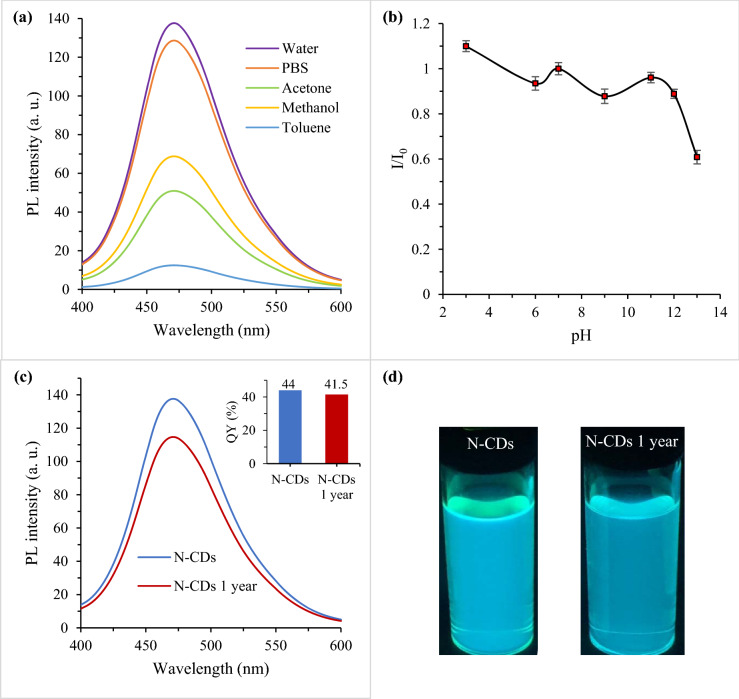
PL spectra of N-CDs as a function of (**a**) solvent (λ_ex_ = 350 nm), (**b**) solution pH, (**c**) freshly synthesized N-CDs suspension (blue curve) and after 1-year storage in dark environment at ambient temperature (red curve) and (**d**) their corresponding optical images under UV (λ_ex_ = 365 nm) exposure. Inset shows the corresponding QYs of the N-CDs.

To further verify the practical applicability of N-CDs, the effect of solvent, solution pH and long-term storage time on the fluorescence behavior were examined. The PL spectra were recorded after dissolving N-CDs in various solvents, including water, Phosphate-buffered saline (PBS), acetone, methanol and toluene. As shown in Fig. 8a, the maximum PL emission was found when N-CDs are dispersed in water, followed sequentially by PBS, methanol, acetone and toluene. The weak PL emission could be due to the photoinduced electron transfer (PET) process between intrinsic nitrogen atoms and surface functional groups of N-CDs^49^. Next, the influence of solution pH on the PL emission is presented in Fig. 8b. It is apparent that the PL emission is enhanced under strong acid conditions, remains unchanged over a wide pH range of 6–12 and weekend in strong alkaline conditions. The pH dependence fluorescence behavior could be ascribed to the protonation/ deprotonation of –OH and –COOH over the N-CDs surface^8^. The spectacular optical characteristics and acidic resistance of N-CDs are of great benefit in the determination of metal ions in acidic environments. Following this, the effect of illumination time and ionic strength on the PL of N-CDs were thoroughly established in our earlier work^40^, and the results indicated that the N–CDs were highly resistant to photobleaching showing great stability under high-salt environment.

Furthermore, no significant drop of PL intensity (Fig. 8c) was found even after being stored 1 year in dark condition at room temperature. The inset in Fig. 8c shows the PL QYs of N-CDs as a function of 1-year storage. In comparison to the freshly prepared N-CDs solution, great PLQY retention of around 97.5% was shown. The corresponding visual photos presented in Fig. 8d agrees well with the results recorded from the PL spectra and QYs calculation, in which N-CDs maintained its performance for almost one year without any aggregation or flocculation. $\xi$ potential value of 1-year N-CDs was also tested (Fig. S4) and found to be − 6.4 mV, which supports the long-term storage stability of N-CDs. The excellent resistance for aging conditions suggests N-CDs as a commercial candidate for biological applications.

### Detection of Fe^3+^ ions in an acidic environment

Nowadays, most of reported N-CDs focuses on heavy metals detection under natural conditions. It is well known that the PL response of N-CDs is unstable in an acidic environment, limiting their function in the field of environmental contamination and metal ions sensing^8^. On top of this, the aforementioned spectroscopic data confirmed that the obtained N-CDs involve –OH, –COO^-^ and –NH functional groups around N-CDs edge. These groups can act as a bridge to bind the nanoprobe with any surrounding analytes^50^. Therefore, we have monitored the PL quenching ability of N-CDs for Fe^3+^ recognition in acidic environment under the condition of pH 3. In order to achieve an efficient sensing performance, the quenching degree of N-CDs towards Fe^3+^ as a function of time intervals were measured using PL spectra (Fig. S5). It is apparent that 50% of the PL emission was declined upon the addition of Fe^3+^ within 1 min, whereas no obvious effect was shown upon rising reaction duration at 465.5 nm. Thus, the following experiments were performed at 1 min, suggesting the fast sensing response of N–CDs for Fe^3+^.

**Figure 9:**
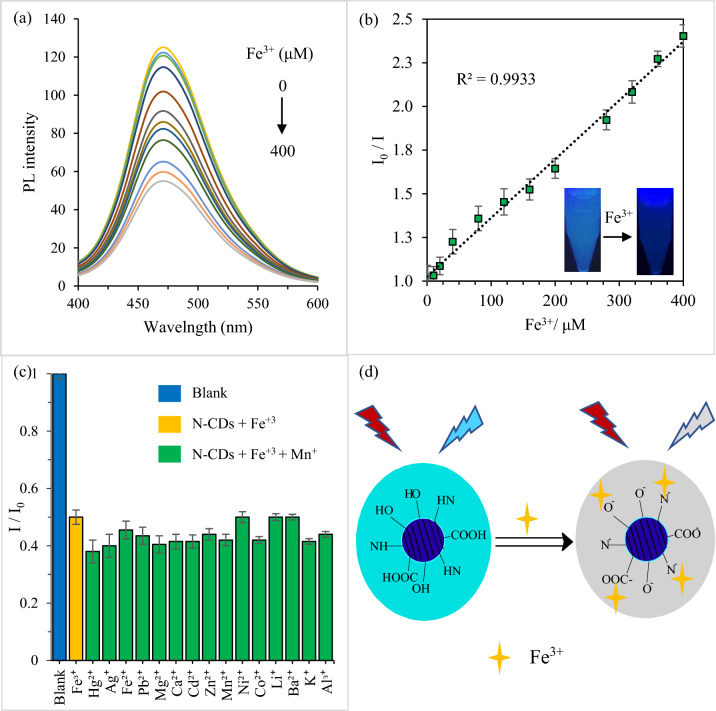
(**a**) PL intensity of N-CDs solution with various concentrations of Fe^3+^ (0, 10, 20, 40, 80, 120, 160, 200, 280, 320, 360 and 400 µM, λ_ex_ = 350 nm). (**b**) the Stern–Volmer plot of N-CDs with increasing the concentration of Fe^3+^. The inset displays the suspension before and after adding Fe^3+^ under 365 nm UV light illumination. (**c**) selectivity of N-CDs for 100 µM of Fe^3+^ and interference of 100 µM other metal ions with 100 µM Fe^3+^. (**d**) schematic representing the PL emission quenching caused by Fe^3+^ chelation.

The sensitivity test was carried out by measuring the PL signal before and after the addition of various concentrations of Fe^3+^ ranging from 0–400 μM. As shown in Fig. 9a, the PL intensity gradually reduced along with the increase in the Fe^3+^ concentration. Additionally, the variation of the PL intensity (I_0_/I) versus Fe^3+^ concentration showed excellent linearity in the range of 1–400 μM, with a correlation coefficient of R^2^ = 0.9933 (Fig. 9b). The visual images are shown in the inset of Fig. 9b represents the PL emission of N-CDs suspension before and after the addition of 100 μM Fe^3+^ ions illuminated with UV-light source (365 nm). It is apparent that the reduction of blue emission by the addition of Fe^3+^ agrees well with the results obtained from PL spectra. The quenching efficiency of the Fe^3+^ can be presented as follows:

4 $$ \frac{{{\text{I}}_{0} - {\text{I}}}}{{{\text{I}}_{0} }} = 1.0258 + 0.0034{ }\left[ {\text{C}} \right] $$

where I and I_0_ represent the PL intensity at 465.5 nm with and without the addition of Fe^3+^ ion, respectively and [C] indicate the Fe^3+^ concentration.

Based on three times signal-to-noise ratio estimations, the LOD was calculated to be 0.14 μM. The LOD of the present sensing system is lower than the maximum permissible level (5.36 µM) stipulated by the World Health Organization (WHO) for Fe^3+^ drinking water. A comparison of detection performance of N-CDs with several reported CD-based Fe^3+^ sensors (Table 7) demonstrated that the present sensing system is superior in terms of sensing interval and LOD.

**Table 7:** Summary of some fluorescent probes for Fe^3+^ sensing.

Probe	Linear range (μM)	LOD (μM)	References
CDs	0–1,500	0.5	^51^
CDs	12.5–100	9.97	^52^
N-CDs	2–25	0.9	^53^
CDs	0–20	0.31	^54^
S, N-CDs	1–370	0.5	^55^
N-CDs	0–2	70	^56^
P, N-CDs	20–200	–	^57^
Rh6G-CDs	0–50	0.727	^58^
N-CDs	0–300	19	^59^
P, N-CDs	1–150	0.33	^60^
N-CDs	1–250	0.52	^61^
CDs	0–50	1.3	^62^
N-CDs	6–200	0.8	^63^
CDs	10–200	1.8	^64^
S, N-CDs	25–500	4	^65^
N–CDs	1–400	0.14	This work

With inherent contamination, metal-ion sensing in real samples exhibits a great challenge to the analytical techniques in terms of sensitivity and selectivity. Thus, to further confirm the feasibility of the proposed sensing system to real samples, the influence of interference of other metal ions on the selective detection of Fe^3+^ was carried out. The PL signal of N-CDs suspension was recorded by mixing 100 μM of Fe^3+^ ions paired and mixed with 100 μM of other metal ions. As shown in Fig. 9c, Fe^3+^ can quench the fluorescence of N-CDs suspension efficiently. Meanwhile, the influence from other coexisting metal ions on the PL intensity is negligible, demonstrating the anti-interference performance of N-CDs for detecting Fe^3+^.

In fact, metal-ion selectivity, binding kinetics, and proton cross-sensitivity are highly dependent on the structure of the chelating groups around the nanoprobe. Thus, the response selectivity of N-CDs with Fe^3+^ among other metal ions could be ascribed to the particular affinity of Fe^3+^ for the oxygen/ nitrogen electron donors, which results in the coordinate covalent bond formation between these atoms (Fig. 9d). These new bonds formed results in the migration of the excited electrons to the vacant d-orbitals of Fe^3+^, leading to non-radiative recombination of the excitons (e–h), and thus result in PL quenching^6,66–69^. Additionally, the ferromagnetic structure of Fe^3+^ ion may also be a key factor causing splitting discrete energy levels of N-CDs, thus, forming channels for overlap of energy levels and inter system crossing (ISC) resulting in PL emission reduction^70^.

To further validate the applicability of the proposed sensing system, the feasibility of N-CDs as a fluorescent probe to detect Fe^3+^ was evaluated in tap water through standard addition techniques. The measurement data of the blank and spiked water samples without and with the addition of Fe^3+^, respectively are shown in Table 8. It could be clearly seen that the spiked recoveries were in the range of 98.02–101.8 whereas the mean relative standard deviation was lower than 2.6%. These results demonstrate the reliability of the developed fluorescent nanosensors for the monitoring of Fe^3+^ in environment.

Based on three times signal-to-noise ratio estimations, the LOD was calculated to be 0.14 μM. The LOD of the present sensing system is lower than the maximum permissible level (5.36 µM) stipulated by the World Health Organization (WHO) for Fe^3+^ drinking water. A comparison of detection performance of N-CDs with several reported CD-based Fe^3+^ sensors (Table 7) demonstrated that the present sensing system is superior in terms of sensing interval and LOD.

**Table 8:** Detection of Fe^3+^ in real water samples.

Spiked (μM)	Detected (μM)	Recovery (%)	RSD^b^ (%, n = 3)
0	ND^a^	–	–
15	15.07	99.67	2.58
30	30.97	101.8	0.84
50	49.5	98.02	1.48

^a^Not detected.

^b^Relative standard deviation.

### Investigation on PL quenching mechanism

In general, two kinds of fluorescent quenching, categorized as dynamic/collisional quenching and static/ ground quenching, are typically observed in the molecular contact between fluorophore and quencher. In dynamic quenching, the excited state of fluorophore returns to the ground state due to the collisions, in which the PL quenching is time and concentration-dependent^71^. Meanwhile, static quenching occurs when a non-fluorescent ground-state complex is formed through the interaction between fluorophore and quencher with a linear dependence quenching^33^. To help understand the turn-off mechanism of N-CDs by Fe^3+^, several spectroscopic techniques with and without Fe^3+^ were measured. In comparison to TEM imaging of pure N-CDs suspension, a significant increment in the size of N-CDs-Fe^3+^ complex was observed (Fig. S6). The size distribution curve was found to be in the range of 7–15 nm for the complex with an average diameter of 8.6 nm, which is bigger than that of N-CDs (3–8; 3.4 nm, respectively). This provides evidence for the aggregation of N-Ds through the formation of N-CDs-Fe^3+^ complexes^72,73^. The possibility of aggregate formation was further supported by Dynamic Light Scattering (DLS) method of N-CD/Fe^3+^ complex which shown an increased hydrodynamic diameter in the range of 50–400 nm with an average size of 285.7 nm (Fig. S7). Following this, ${\upxi }$ potential value of 5.08 eV after the addition of 100 μM Fe^3+^ was observed (Fig. S8). In comparison to ${\upxi }$ potential of N-CDs (Fig. S3), this rise in ${\upxi }$ potential suggested that the charges of N-CDs were neutralized with the positive charges of Fe^3+^ due to the electrostatic effect.

Moreover, the temperature-dependent PL intensity of the Fe^3+^ chelated N-CDs was also determined. As shown in Fig. S9, the PL intensity of the Fe^3+^ treated N-CDs gradually decreases with increasing temperature, which is a typical case for the static quenching and hence confirm the static quenching process. More specifically, rise in the temperature decline the molecular interactions between Fe^3+^ and N-CDs. After that, dissociation of the weakly bound nanocomplexes occurs, leading to the presence of a small fraction of statically quenched undissolved nanocomplexes in the medium^66^.

FTIR spectrum was conducted to further confirm the nanocomplex formation. As shown in Fig. S10, significant red-shifting of the wavenumbers and great increments of the characteristic peaks, particularly at C–N, C = O, C–O and C–O–C, were observed. This reveals the plausible involvement of the carboxylic group of N-CDs along with N containing groups in Fe^3+^ complexation. As a result, the variations observed in morphology, $\xi$ potential, temperature and FTIR analysis confirmed the formation of the nonfluorescent complex between surface functional groups of N-CDs and Fe^3+^ ions, resulting in the PL quenching of N-CDs.

### Fluorescent ink application

**Figure 10:**
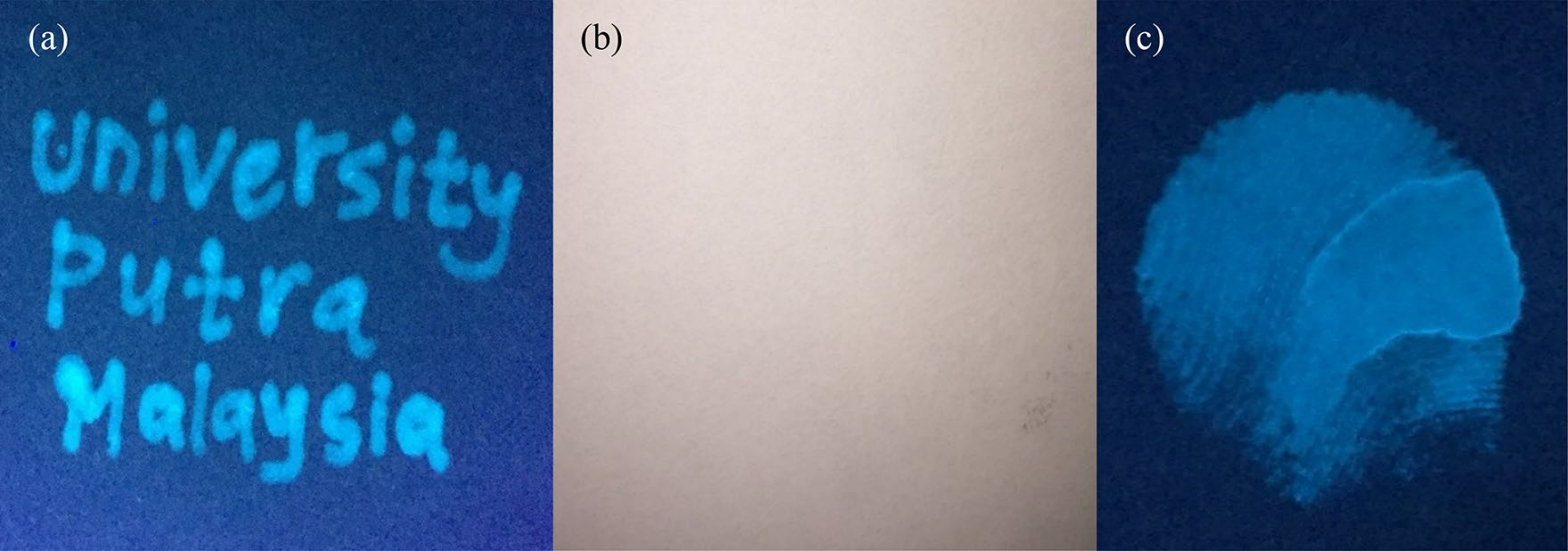
Text written on commercial filter paper using N-CDs invisible ink under (**a**) UV light and (**b**) daylight. (**c**) N-CDs based fingerprints under UV-light.

The N-CDs solution was further utilized as a fluorescent ink on the basis of their high solubility, great photostability, and intense PL capacity. To evaluate this, the aqueous solution of N-CDs was conveniently injected into a drawing pen (Fig. S11) and facilitated for writing several words on the commercial paper. As presented in Fig. 10a, the handwriting words “University Putra Malaysia” on filter paper can be clearly seen under the excitation of 365 nm UV light (exhibited intense blue emission). After 6 months of storage time, the PL intensity of the hand-written image has no significant change when stored under ambient conditions (Fig. S12), suggesting that N-CDs have long-term PL stability. In addition, the hand-written words are totally colorless and invisible under daylight (Fig. 10b), which provides a guarantee of using N-CDs as a promising candidate in document labeling, security purposes, printing, stamps, and so on. Further, N-CDs can be also used as ink pads to form human fingerprints (Fig. 10c) because of their great low/non-toxic nature, long PL lasting and easily washed out with water. In general, the bright PL emission with high PLQY, long shelf-life, high resistance to photobleaching, ecofriendly and low preparation cost suggest N-CDs as an excellent competitor to the existing fluorescent pens^53,74–76^.

## Conclusions

Highly luminescence, eco-friendly and water-soluble N-CDs have been successfully synthesized from CMC of EFB with the incorporation of LPEI as surface passivation agent/ N-doping source through one-pot HTC route. The ultra-small highly luminescence N-CDs were rapidly fabricated without the necessity of using harsh toxic chemicals or long-time synthesis procedures. The as-formed N-CDs with an average size of 3.4 nm exhibited bright and stable emission even after a period of 1-year storage at room temperature. According to ANOVA data, the experimental results were in good agreement with predicted values, in which synthesis duration was found to be the most significant factor that affects the QY and followed by the temperature and LPEI weight. Under optimal synthesis conditions, QY of around 44% could be obtained. The as-produced N-CDs were applied as a fluorescent nanoprobe for Fe^3+^ detection, in which a good linear correlation in the concentration range of 1–400 μM with a detection limit of 0.14 μM were obtained at pH 3. N-CDs were further employed as invisible ink for data encryption and forming fingerprints. It is believed that the production of highly fluorescent N-CDs from these renewable resources in a significantly short-duration could emerge as a new finding.

## Supplementary information

Supplementary information.
