# Supplementary material for: Fluorescent recognition of Fe3+ in acidic environment by enhanced-quantum yield N-doped carbon dots: optimization of variables using central composite design
Source: Sci Rep. 2020 Jul 16;10:11710. doi: 10.1038/s41598-020-68390-8 (PMC7366660; doi:10.1038/s41598-020-68390-8)
Supplement: Supplementary file 1 — Supplementary information. [file 41598_2020_68390_MOESM1_ESM.pdf]

Fluorescent recognition of Fe^{3+} in an acidic environment by enhanced-quantum yield N-doped carbon dots: optimization of variables using central composite design

Mohammed Abdullah Issa [1,*], Zurina Z. Abidin [1,*], Shafreeza Sobri [1], Suraya Rashid [1], Mohd Adzir Mahdi [2], Nor Azowa Ibrahim [3]

[1]Department of Chemical and Environmental Engineering, Faculty of Engineering, Universiti Putra Malaysia, 43400 UPM Serdang, Selangor, Malaysia
[2]Department of Computer & Communications Systems Engineering, Faculty of Engineering, Universiti Putra Malaysia, 43400 UPM Serdang, Selangor, Malaysia
[3]Department of Chemistry, Faculty of Science, Universiti Putra Malaysia, 43400 Serdang, Selangor, Malaysia

Table S1. A summary of different biomass used for production of CDs via hydrothermal route

Precursors	Temperature (°C)	Time consumed (h)	QY (%)	Refs
Apple juice	150	12	4.27	[1]
Grass	180	3	6.2	[2]
Lemon waste peels	200	12	14	[3]
Kitchen waste	180	6	3.1	[4]
Bagasse	180	3	12.3	[5]
Pomelo peel waste	200	3	6.9	[6]
Sugarcane molasses	250	12	5.8	[7]
Glucose, PEI	150	12	2.86	[8]
Xylan, NH_4OH	200	12	16	[9]
Microcrystalline cellulose, EDA	240	12	51	[10]
Cellulose, $(NH_4)_2CO_3$	180	12	7.6	[11]
Cellulose,urea, $Al(No_3)_3$	210	12	28.7	[12]
Cellulose, urea	180	72	21	[13]
CMC EFB, EDA	270	6	22.99	[14]
CMC EFB, LPEI	260	2	44	This work

Table S2. Optimization studies of N-CDs using RSM design

Synthesis method	Type of design	Variables optimized	Most influential variable	Suggested model	Ref
HTC	Box-Behnken	Temperature, time and tris[a] weight	Time and tris weight	Cubic	[15]
HTC	CCD	Time, temperature, and (NH_4) HCO_3[b] weight	Temperature	Reduced cubic	[16]
Microwave-assisted-pyrolysis	CCD	Time, EDA[c]/citric acid ratio, and citric acid concentration	EDA/citric acid ratio	Quadratic	[17]
HTC	CCD	Temperature, time and EDA[d] weight	Temperature	Quadratic	[14]

[a]Tris(hydroxymethyl)aminomethane, [b]ammonium bicarbonate, [c]ethylendiamine, [d]Ethylendiamine.

Statistical analysis

The evaluation of RSM model was performed using statistical analysis of central composite design. Typically, three major tests, including significant of terms, regression model and lack-of-fit test were used to evaluate the adequacy and reliability of a model. Multiple polynomial models including Linear, 2FI (two factorial), quadratic, and cubic models were fitted according to the experimental data for obtaining the final regression equation using analysis of variance (ANOVA). Probability values F-value and P-value were used to determine the most significant variables on the response based on significance of terms. Higher F-value along with lower P-value (normally less than 0.05) of the model terms are considered to have significant influence on the response. The lack-of-fit test describes the unsuccessful model to explain the experimental data that are excluded from the regression. The significant model should have non-significant lack-of-fit, which indicates that the residual error has not exceeded pure error and hence the model is significant. To verify the fitness of the model, determination coefficient (R^2), adjusted R^2, predicted R^2, and predicted error sum of squares (PRESS) are used. The value of R^2 shows the percentage of the total variations in experimentation which are not explained by the model whereas adjusted R^2 is the variation of the mean described by the model in which the number of factors of the data set is considered. Normally, a good model should have R^2 value of close to one, suggesting that better correlation between actual data and the empirical model would be obtained. The reproducibility of the model was assessed based on the Coefficient of Variation (CV) and predicted error sum of squares (PRESS). Typically, the CV $\leq$ 10 % and PRESS of greater than 4 confirms acceptable reproducibility for the model. The influence of individual factors and their interaction effect on the response were further evaluated using three-dimension plots. the suggested model was finally used to identify and validate the optimum conditions of each variable for obtaining highest efficiency of the response (i. e. quantum yield).

Table S3. Quantum yield of the obtained N-CDs, with λex = 350 nm

Run number	Optical density (OD)	Integrated emission intensity (I)	Quantum yield (QY)

1	0.04	11245.7	13.98
2	0.04	16550.5	20.58
3	0.04	14082.5	17.51
4	0.04	14985.5	18.63
5	0.04	10350.1	12.87
6	0.04	12620.2	15.69
7	0.04	12620.7	15.69
8	0.04	14485.5	18.01
9	0.04	20347.4	25.30
10	0.04	35389.1	44.00
11	0.04	12591.7	15.66
12	0.04	31550.2	39.23
13	0.04	28277.4	35.16
14	0.04	21287.4	26.47
15	0.04	30718.6	38.20
16	0.04	31040	38.60
17	0.04	30478.6	37.90
18	0.04	29758.2	37.00
Quinine sulfate	0.033	35828.9	54 (known)

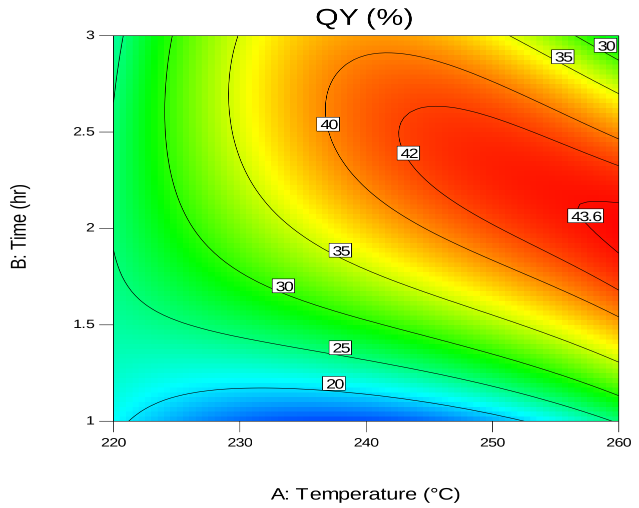

Fig. S1. Contour plots showing the influence of synthesis parameters on the QY as predicted by RSM model with LPEI weight held constant at 1%

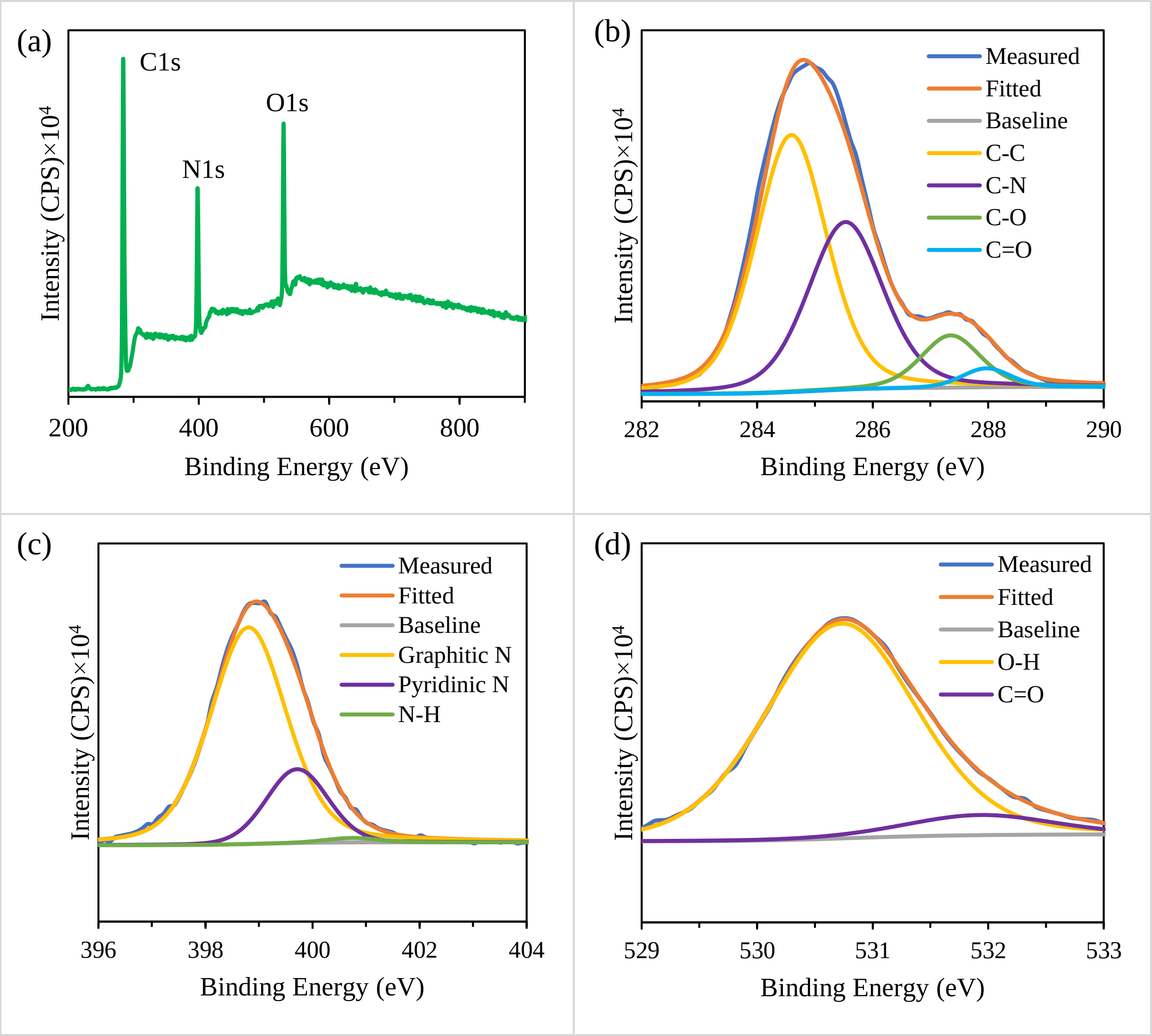

Fig. S2. (a) XPS full survey spectra of N–CDs. The high-resolution XPS spectra of (b) C1s, (c) N1s and (d) O1s

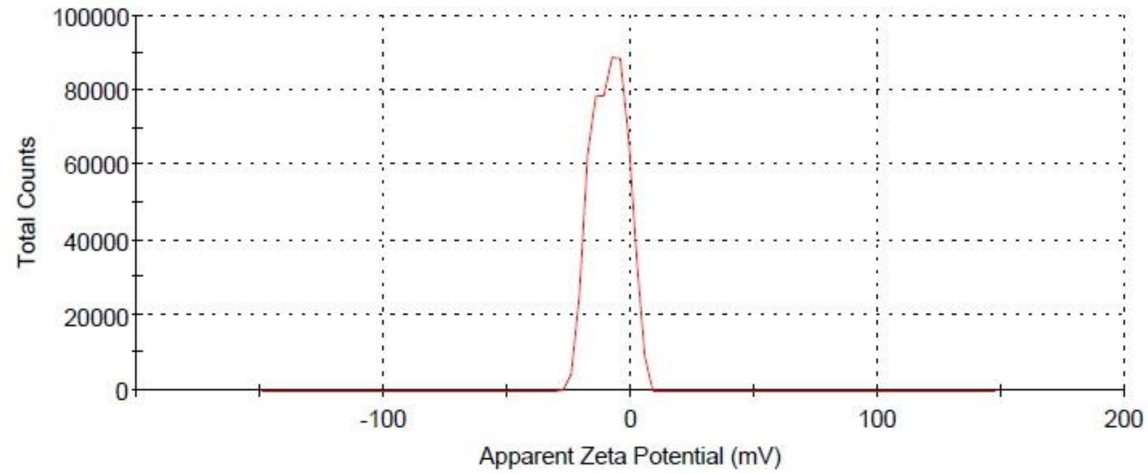

Fig. S3. Zeta potential of N-CDs suspension

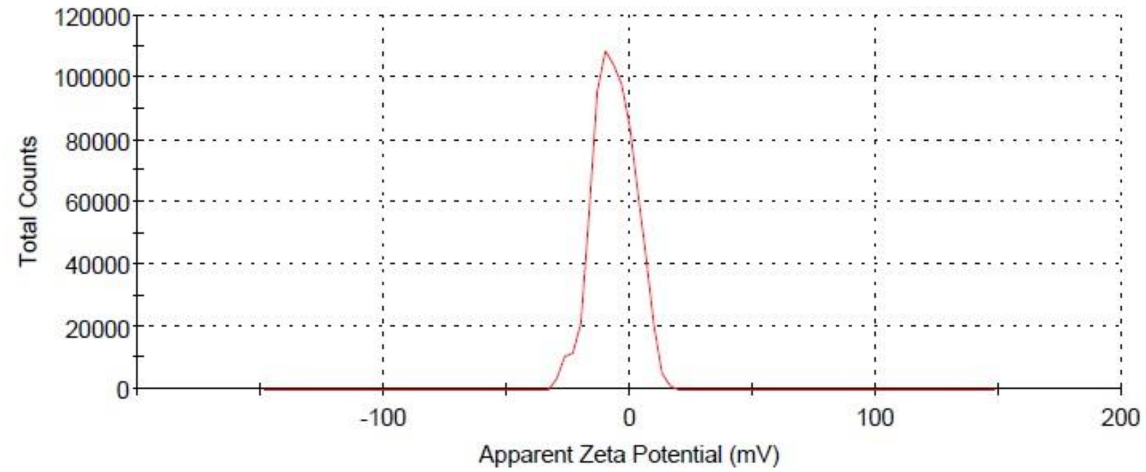

Fig. S4. Zeta potential of N-CDs suspension after 1 year of storage in ambient condition

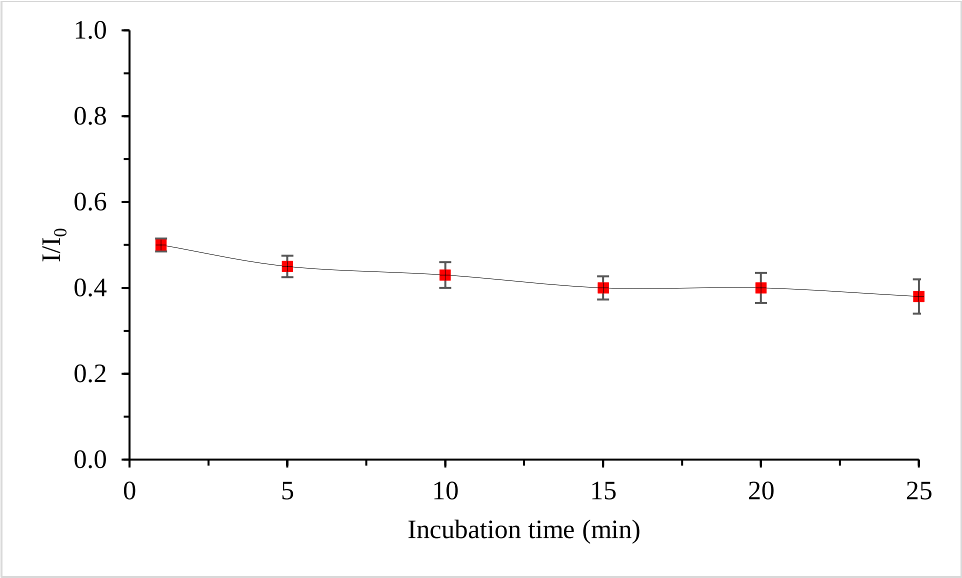

Fig. S5. Effect of reaction time on the relative fluorescence intensity (I/I_0) of N-CDs suspension with and without Fe^{3+}

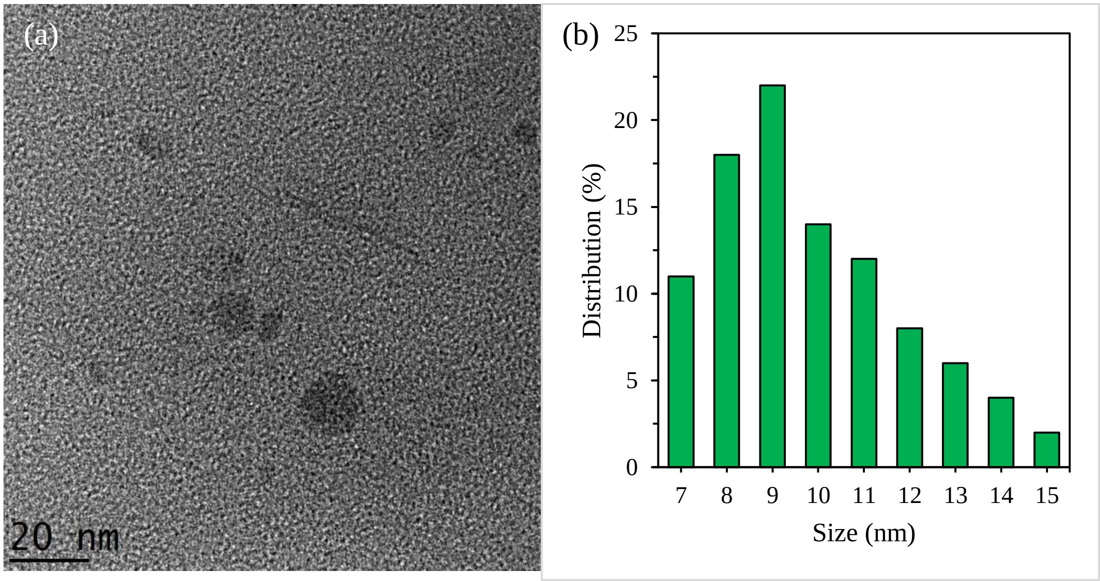

Fig. S6. TEM images (a) and size distribution (b) of N-CDs after the addition of Fe^{3+}

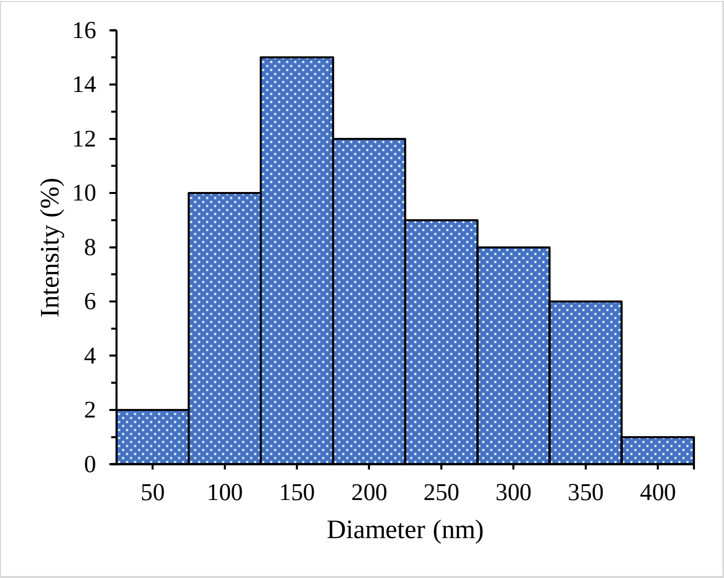

Fig. S7. DLS size distribution of N-CDs suspension after the addition of Fe^{3+}

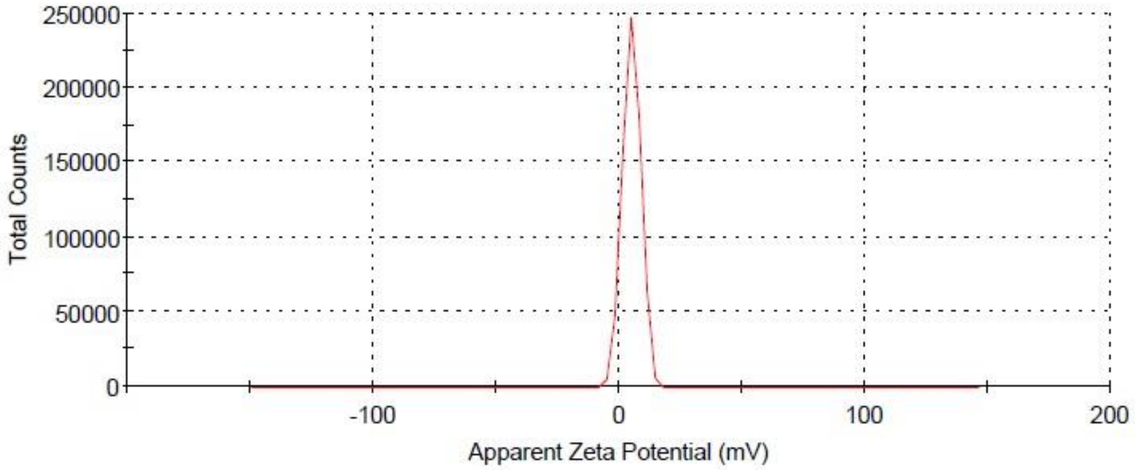

Fig. S8. Zeta potential of N-CDs suspension after the addition of Fe^{3+}

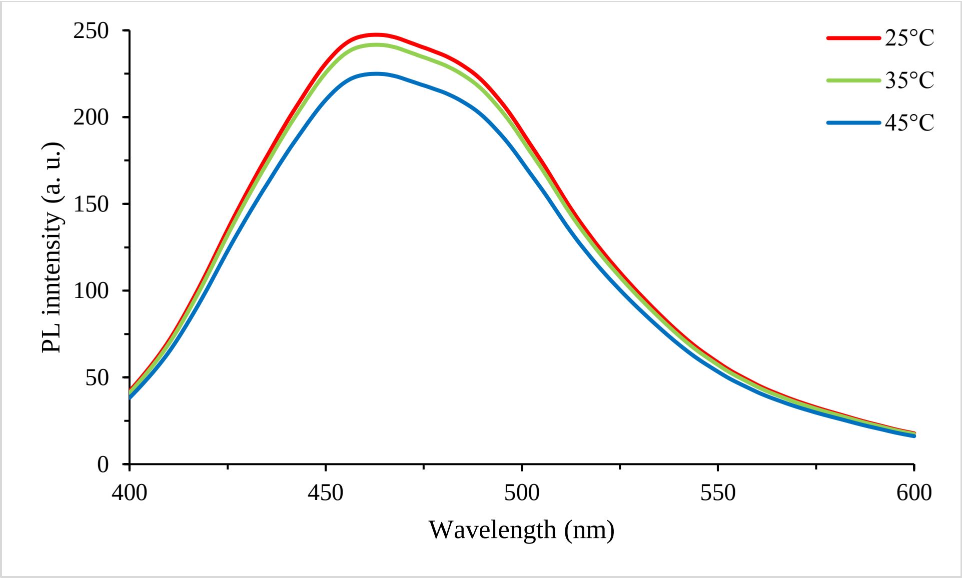

Fig. S9. Temperature-dependent PL intensity of the Fe^{3+} chelated N-CDs

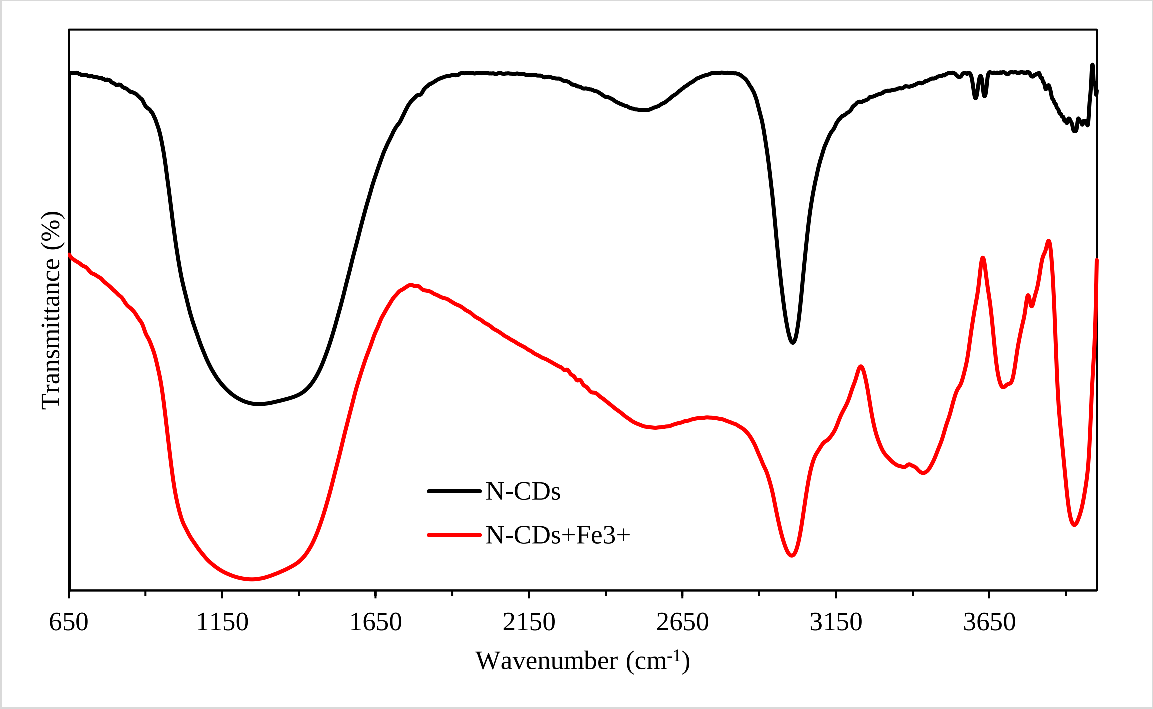

Fig. S10. FTIR of N-CDs before (black trace) and after (red trace) the addition of Fe^{3+}

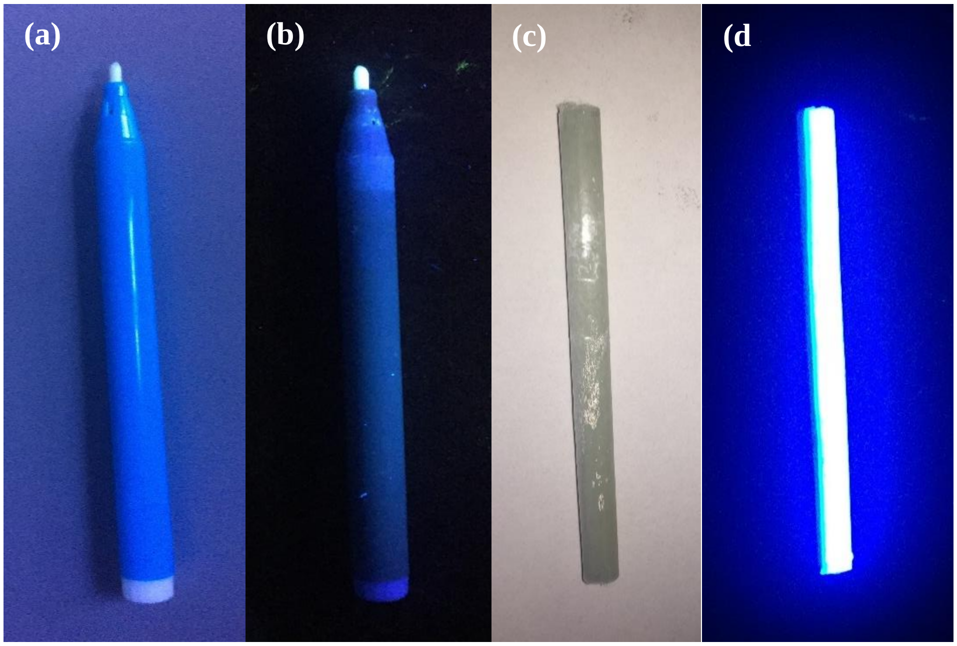

Fig. S11. Sketch pen containing aqueous dispersion N-CDs and internal part filled with N-CDs (a, c) under day light and (b, d) under UV light

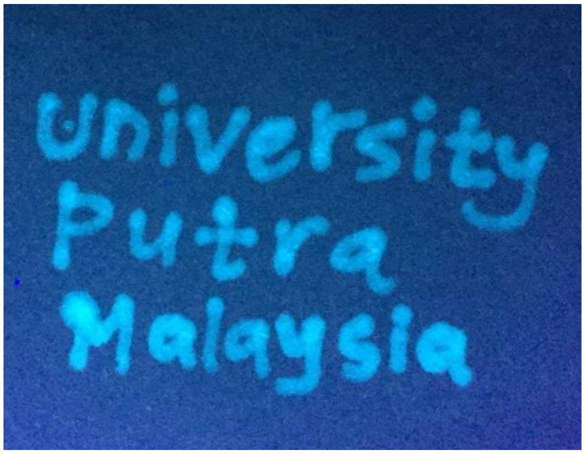

Fig. 12. Reproducability of hand-written images using N-CDs ink under 365 nm UV light after 6 months of storage under room temperature

Reference

[1] V. N. Mehta, S. Jha, H. Basu, R. K. Singhal, and S. K. Kailasa, "One-step hydrothermal approach to fabricate carbon dots from apple juice for imaging of mycobacterium and fungal cells," *Sensors Actuators, B Chem.*, vol. 213, pp. 434–

443, 2015.

[2] X. Qin, W. Lu, A. M. Asiri, A. O. Al-Youbi, and X. Sun, “Microwave-assisted rapid green synthesis of photoluminescent carbon nanodots from flour and their applications for sensitive and selective detection of mercury(II) ions,” *Sensors Actuators, B Chem.*, vol. 184, pp. 156–162, 2013.

[3] V. A. Online, N. Singh, S. Choudhary, and R. K. Gupta, “RSC Advances,” 2016.

[4] J. Xu, T. Lai, Z. Feng, and X. Weng, “Formation of fluorescent carbon nanodots from kitchen wastes and their application for detection of Fe3+,” *Luminescence*, vol. 30, no. 4, pp. 420– 424, 2014.

[5] F. Du, M. Zhang, X. Li, J. Li, and X. Jiang, “Economical and green synthesis of bagasse-derived fluorescent carbon dots for biomedical applications,” *Nanotechnology*, vol. 315702, 2014.

[6] W. Lu *et al.*, “Economical, green synthesis of fluorescent carbon nanoparticles and their use as probes for sensitive and selective detection of mercury(II) ions,” *Anal. Chem.*, vol. 84, no. 12, pp. 5351–5357, 2012.

[7] G. Huang *et al.*, “sugarcane molasses : synthesis , properties , and,” *RSC Adv.*, vol. 7, pp. 47840–47847, 2017.

[8] Q. Dou, X. Fang, S. Jiang, P. L. Chee, T. C. Lee, and X. J. Loh, “Multi-functional fluorescent carbon dots with antibacterial and gene delivery properties,” *RSC Adv.*, vol. 5, no. 58, pp. 46817–46822, 2015.

[9] Z. Liang, L. Zeng, X. Cao, Q. Wang, X. Wang, and R. Sun, “Sustainable carbon quantum dots from forestry and agricultural biomass with amplified photoluminescence by simple NH 4 OH passivation,” *J. Mater. Chem. C*, vol. 2, no. 45, pp. 9760–9766, 2014.

[10] P. Wu, W. Li, Q. Wu, Y. Liu, and S. Liu, “Hydrothermal synthesis of nitrogen-doped carbon quantum dots from microcrystalline cellulose for the detection of Fe 3+ ions in an acidic environment,” *RSC Adv.*, vol. 7, no. 70, pp. 44144–44153, 2017.

[11] J. Wang *et al.*, “WITHDRAWN: One-pot simple green synthesis of water-soluble cleaner fluorescent carbon dots from cellulose and its sensitive detection of iron ion,” *J. Clean. Prod.*, 2017.

[12] S. Jayaweera, K. Yin, X. Hu, and W. J. Ng, “Fluorescent N/Al Co-Doped Carbon Dots from Cellulose Biomass for Sensitive Detection of Manganese (VII),” *J. Fluoresc.*, 2019.

[13] P. Shen, J. Gao, J. Cong, Z. Liu, C. Li, and J. Yao, “Synthesis of Cellulose-Based Carbon Dots for Bioimaging,” *ChemistrySelect*, vol. 1, no. 7, pp. 1314–1317, 2016.

[14] M. Abdullah Issa *et al.*, “Fabrication, characterization and response surface method optimization for quantum efficiency of fluorescent nitrogen-doped carbon dots obtained from carboxymethylcellulose of oil palms empty fruit bunch,” *Chinese J. Chem. Eng.*, 2019.

[15] Y. Jiliang, “Optimized preparation of nitrogen-doped carbon dots by response surface methodology and application in Cd2+ detection,” *Fullerenes, Nanotub. Carbon Nanostructures*, vol. 27, pp. 233–239, 2019.

[16] A. Barati, M. Shamsipur, E. Arkan, L. Hosseinzadeh, and H. Abdollahi, “Synthesis of biocompatible and highly photoluminescent nitrogen doped carbon dots from lime: Analytical applications and optimization using response surface methodology,” *Mater. Sci. Eng. C*, vol. 47, pp. 325–332, 2015.

[17] J. Liang, S. Kavadiya, M. Y. Berezin, P. Biswas, and N. Ravi, “Optimizing the Synthesis of Red-Emissive Nitrogen-Doped Carbon Dots for Use in Bioimaging,” *ACS Appl. Nano Mater*, vol. 1, no. 7, pp. 3682–3692, 2018.
